# The selective peroxisome proliferator-activated receptor alpha modulator (SPPARMα) paradigm: conceptual framework and therapeutic potential: A consensus statement from the International Atherosclerosis Society (IAS) and the Residual Risk Reduction Initiative (R3i) Foundation

**DOI:** 10.1186/s12933-019-0864-7

**Published:** 2019-06-04

**Authors:** Jean-Charles Fruchart, Raul D. Santos, Carlos Aguilar-Salinas, Masanori Aikawa, Khalid Al Rasadi, Pierre Amarenco, Philip J. Barter, Richard Ceska, Alberto Corsini, Jean-Pierre Després, Patrick Duriez, Robert H. Eckel, Marat V. Ezhov, Michel Farnier, Henry N. Ginsberg, Michel P. Hermans, Shun Ishibashi, Fredrik Karpe, Tatsuhiko Kodama, Wolfgang Koenig, Michel Krempf, Soo Lim, Alberto J. Lorenzatti, Ruth McPherson, Jesus Millan Nuñez-Cortes, Børge G. Nordestgaard, Hisao Ogawa, Chris J. Packard, Jorge Plutzky, Carlos I. Ponte-Negretti, Aruna Pradhan, Kausik K. Ray, Željko Reiner, Paul M. Ridker, Massimiliano Ruscica, Shaukat Sadikot, Hitoshi Shimano, Piyamitr Sritara, Jane K. Stock, Ta-Chen Su, Andrey V. Susekov, André Tartar, Marja-Riitta Taskinen, Alexander Tenenbaum, Lale S. Tokgözoğlu, Brian Tomlinson, Anne Tybjærg-Hansen, Paul Valensi, Michal Vrablík, Walter Wahli, Gerald F. Watts, Shizuya Yamashita, Koutaro Yokote, Alberto Zambon, Peter Libby

**Affiliations:** 1 R3i Foundation, Picassoplatz 8, 4010 Basel, Switzerland; 2 Hospital Israelita Albert Einstein, and Lipid Clinic, Heart Institute (InCor) University of Sao Paulo Medical School Hospital, Sao Paulo, Brazil; 3 Unidad de Investigacion de Enfermedades Metabolicas, Department of Endocrinolgy and Metabolism, Instituto Nacional de Ciencias Médicas y Nutrición Salvador Zubirán, Mexico City, Mexico; 4 Tecnologico de Monterrey, Escuela de Medicina y Ciencias de la Salud, Monterrey, Mexico; 5 Center for Interdisciplinary Cardiovascular Sciences and Center for Excellence in Vascular Biology, Division of Cardiovascular Medicine and Channing Division of Network Medicine, Brigham and Women’s Hospital, Harvard Medical School, Boston, MA USA; 6 Department of Clinical Biochemistry, Sultan Qaboos University Hospital, Muscat, Oman; 7 Department of Neurology and Stroke Center, Paris-Diderot-Sorbonne University, Paris, France; 8 Lipid Research Group, School of Medical Sciences, University of New South Wales, Sydney, NSW Australia; 9 IIIrd Dept Int. Med, Center for Preventive Cardiology, 3rd Internal Medicine Clinic, University General Hospital and Charles University, Prague, Czech Republic; 10 Department of Pharmacological and Biomolecular Sciences, Università Degli Studi di Milano, Milan, Italy; 11 Centre de recherche sur les soins et les services de première ligne-Université Laval du CIUSSS de la Capitale-Nationale, Department of Kinesiology, Faculty of Medicine, Université Laval, Québec, QC Canada; 12 INSERM, CHU Lille, U1171-Degenerative & Vascular Cognitive Disorders, University of Lille, Faculty of Pharmacy, University of Lille, UDSL, Lille, France; 13 Division of Endocrinology, Metabolism and Diabetes, Department of Medicine, University of Colorado School of Medicine, Anschutz Medical Campus, Aurora, CO USA; 14 Laboratory of Lipid Disorders, National Cardiology Research Center, Moscow, Russian Federation; 15 Lipid Clinic, Point Médical and Department of Cardiology, CHU Dijon-Bourgogne, Dijon, France; 16 Columbia University Vagelos College of Physicians and Surgeons, New York, USA; 17 Division of Endocrinology and Nutrition, Cliniques Universitaires St-Luc and Institut de Recherche Expérimentale et Clinique (IREC), Université Catholique de Louvain, Brussels, Belgium; 18 Division of Endocrinology and Metabolism, Department of Internal Medicine, Jichi Medical University, Shimotsuke, Japan; 19 OCDEM, University of Oxford and the NIHR Oxford Biomedical Research Centre, OUH Foundation Trust, Churchill Hospital, Oxford, UK; 20 Laboratory for System Biology and Medicine Research Center for Advanced Science and Technology, The University of Tokyo, Tokyo, Japan; 21 Deutsches Herzzentrum München, Technische Universitat München, Germany; DZHK (German Centre for Cardiovascular Research), Partner Site Munich Heart Alliance, Munich, Germany; 22 Institute of Epidemiology and Medical Biometry, University of Ulm, Ulm, Germany; 23 Mass Spectrometry Core facility of West Human Nutrition Research Center (CRNHO), Hotel Dieu Hospital, Nantes, France; 24 Inra, UMR 1280, Physiologie des Adaptations Nutritionnelles, Nantes, France; 25 Department of Endocrinology, Metabolic diseases and Nutrition, G and R Laennec Hospital, Nantes, France; 26 Department of Internal Medicine, Seoul National University Bundang Hospital and Seoul National University College of Medicine, Seongnam, Republic of Korea; 27 DAMIC Medical Institute/Rusculleda Foundation for Research, Córdoba, Argentina; 28 Cardiology Department, Córdoba Hospital, Córdoba, Argentina; 29 Ruddy Canadian Cardiovascular Genetics Centre, University of Ottawa Heart Institute, Ottawa, Canada; 30 Internal Medicine, Lipids Unit, Gregorio Marañón University Hospital, Madrid, Spain; 31 Department of Medicine, School of Medicine, Universidad Complutense de Madrid, Madrid, Spain; 32 Instituto de Investigaciones Sanitarias Gregorio Marañón, Madrid, Spain; 33 Department of Clinical Biochemistry, Herlev and Gentofte Hospital, Copenhagen University Hospital, Herlev, Denmark; 34 The Copenhagen General Population Study, Herlev and Gentofte Hospital, Copenhagen University Hospital, Herlev, Denmark; 35 Faculty of Health and Medical Sciences, University of Copenhagen, Copenhagen, Denmark; 36 National Cerebral and Cardiovascular Center, Suita, Osaka Japan; 37 Institute of Cardiovascular and Medical Sciences, University of Glasgow, Glasgow, UK; 38 Cardiovascular Medicine, Brigham and Women’s Hospital, Harvard Medical School, Boston, MA USA; 39 Unidad de Prevención Cardiometabólica Cardiocob. Servicio de Cardiología Hospital el Pino Santiago de Chile, Sociedad Inter Americana de Cardiología SIAC Chairman Cardiovascular Prevention Comite, Santiago de Chile, Chile; 40 Division of Cardiovascular Medicine, VA Boston Medical Center, Boston, MA USA; 41 Division of Preventive Medicine, Brigham and Women’s Hospital, Harvard Medical School, Boston, MA USA; 42 Imperial Centre for Cardiovascular Disease Prevention, Department of Primary Care and Public Health, Imperial College London, London, UK; 43 Department of Internal Medicine, University Hospital Centre Zagreb, School of Medicine, Zagreb University, Kispaticeva 12, Zagreb, Croatia; 44 Division of Cardiovascular Medicine and Center for Cardiovascular Disease Prevention, Brigham and Women’s Hospital, Harvard Medical School, Boston, MA USA; 45 Department of Endocrinology/Diabetology, Jaslok Hospital and Research Centre, Mumbai, India; 46 Department of Internal Medicine (Endocrinology and Metabolism), Faculty of Medicine, University of Tsukuba, Ibaraki, 305-8575 Japan; 47 Department of Medicine, Ramathibodi Hospital, Mahidol University, Bangkok, Thailand; 48 Departments of Internal Medicine and Environmental and Occupational Medicine, National Taiwan University; Institute of Occupational Medicine and Industrial Hygiene, National Taiwan University College of Public Health, Taipei, Taiwan; 49 Faculty of Clinical Pharmacology and Therapeutics, Academy for Postgraduate Continuous Medical Education, Moscow, Russian Federation; 50 Faculté de Pharmacie de Lille, Lille, France; 51 Research Program for Clinical and Molecular Metabolism, Faculty of Medicine, University of Helsinki and Clinical Research Institute, HUCH Ltd., Helsinki, Finland; 52 Sackler Faculty of Medicine, Tel Aviv University, 6997801 Tel Aviv, Israel; 53 Cardiac Rehabilitation Institute, Sheba Medical Center, 5265601 Tel Hashomer, Israel; 54 Department of Cardiology, Faculty of Medicine, Hacettepe University, Ankara, Turkey; 55 Department of Medicine & Theraputics, The Chinese University of Hong Kong, Hong Kong, Hong Kong; 56 Department of Clinical Biochemistry, Rigshospitalet; Copenhagen University Hospital, Faculty of Health and Medical Sciences, University of Copenhagen, Copenhagen, Denmark; 57 The Copenhagen General Population Study, Herlev and Gentofte Hospital, Herlev, Denmark; 58 Department of Endocrinology, Diabetology and Nutrition, Jean-Verdier Hospital (AP-HP), Paris 13 University, Sorbonne Paris Cité, CRNH-IdF, CINFO, 93140 Bondy, France; 59 3rd Department of Medicine, 1st Faculty of Medicine of Charles University and General University Hospital in Prague, Prague, Czech Republic; 60 Lee Kong Chian School of Medicine, Nanyang Technological University Singapore, Clinical Sciences Building, 11 Mandalay Road, Singapore, 308232 Singapore; 61 Center for Integrative Genomics, Université de Lausanne, Le Génopode, CH-1015 Lausanne, Switzerland; 62 Institut National de La Recherche Agronomique (INRA), UMR1331 ToxAlim, Toulouse, France; 63 Lipid Disorders Clinic, Department of Cardiology, Royal Perth Hospital, School of Medicine, University of Western Australia, Perth, Australia; 64 Rinku General Medical Center, Izumisano, Osaka Japan; 65 Department of Community Medicine, Osaka University Graduate School of Medicine, Suita, Osaka Japan; 66 Department of Cardiovascular Medicine, Osaka University Graduate School of Medicine, Suita, Osaka Japan; 67 Department of Endocrinology, Hematology and Gerontology, Clinical Cell Biology and Medicine, Chiba University Graduate School of Medicine, Chiba, Japan; 68 Department of Medicine-DIMED, University of Padua, Padua, Italy; 69 Division of Cardiovascular Medicine, Department of Medicine, Brigham and Women’s Hospital, Harvard Medical School, Boston, MA USA

**Keywords:** Residual cardiovascular risk, Visceral obesity, Diabetes, Atherogenic dyslipidemia, Triglycerides, Remnant cholesterol, Selective peroxisome proliferator-activated receptor alpha modulator, SPPARMalpha, Pemafibrate (K-877), Inflammation, PROMINENT

## Abstract

In the era of precision medicine, treatments that target specific modifiable characteristics of high-risk patients have the potential to lower further the residual risk of atherosclerotic cardiovascular events. Correction of atherogenic dyslipidemia, however, remains a major unmet clinical need. Elevated plasma triglycerides, with or without low levels of high-density lipoprotein cholesterol (HDL-C), offer a key modifiable component of this common dyslipidemia, especially in insulin resistant conditions such as type 2 diabetes mellitus. The development of selective peroxisome proliferator-activated receptor alpha modulators (SPPARMα) offers an approach to address this treatment gap. This Joint Consensus Panel appraised evidence for the first SPPARMα agonist and concluded that this agent represents a novel therapeutic class, distinct from fibrates, based on pharmacological activity, and, importantly, a safe hepatic and renal profile. The ongoing PROMINENT cardiovascular outcomes trial is testing in 10,000 patients with type 2 diabetes mellitus, elevated triglycerides, and low levels of HDL-C whether treatment with this SPPARMα agonist safely reduces residual cardiovascular risk.

**Electronic supplementary material:**

The online version of this article (10.1186/s12933-019-0864-7) contains supplementary material, which is available to authorized users.

## Preamble: why we need this consensus

Atherosclerotic cardiovascular disease (ASCVD) presents a growing global health challenge. Over the last 20 years, chronic lifestyle-related diseases such as visceral obesity, type 2 diabetes mellitus (T2DM), and non-alcoholic fatty liver disease (NAFLD) have exacerbated the burden of death and disability due to ASCVD. While this burden affects all regions, it presents a particular threat in low- and middle-income countries, which have the largest populations affected by obesity and diabetes [1–3]. Furthermore, escalation in the prevalence of NAFLD in these regions, particularly the Middle East and Latin America, has contributed to this increasing ASCVD burden independent of traditional risk factors [4, 5].

Atherogenic dyslipidemia, however, remains a major unmet clinical need in such populations. Elevated plasma triglycerides (TG), with or without low levels of high-density lipoprotein cholesterol (HDL-C), offer a key modifiable component of this common pattern of dyslipidemia, especially in those with insulin resistant conditions such as T2DM. After statins, guidelines recommend peroxisome proliferator-activated receptor alpha (PPARα) agonists—fibrates—for management of hypertriglyceridemia [6]. However, these agents have limitations, most importantly due to pharmacokinetic interactions, such as increased risk of myopathy with statins for gemfibrozil [7], or side effects, which include reversible elevation in serum creatinine (with fenofibrate), as well as liver enzyme elevation [8–10]. Hence, there is a clear need for new therapeutic options.

Is it possible to selectively modify the pharmacological characteristics of a PPARα agonist to improve the profile of beneficial effects and address known safety issues associated with fibrate treatment? And, if this is feasible, would this represent a novel therapeutic class? This Joint Consensus Panel from the International Atherosclerosis Society (IAS) and the Residual Risk Reduction Initiative (R3i) evaluated these questions in the context of evidence for the first of the selective peroxisome proliferator-activated receptor alpha modulators (SPPARMα). Box 1 delineates the search strategy and selection criteria for studies that informed this statement.

Box 1. Search strategy and selection criteria References were identified through searches of PubMed for articles published from 2000, by the use of the terms ‘selective peroxisome proliferator-activated receptor alpha’; ‘nuclear receptor’; ‘fibrate’; ‘remnant cholesterol’; ‘cardiovascular risk’; ‘residual risk’; ‘triglyceride-rich lipoproteins’; ‘non-alcoholic fatty liver disease’; ‘pemafibrate (K-877)’; in combination with the term’ diabetes‘, ‘obesity’, ‘atherosclerotic cardiovascular disease’ and ‘non-alcoholic fatty liver disease’. Relevant articles were also identified through searches of the reference lists of the identified literature. Articles resulting from these searches and relevant references cited in those articles were reviewed. Only articles published in English were included.

## Residual vascular risk: a key therapeutic concept

Despite guideline-recommended treatment of ASCVD risk, including antihypertensive and high-intensity statin therapy, or antiaggregant agents, high-risk patients, especially those with established ASCVD, continue to experience cardiovascular events [11, 12]. This residual vascular risk, particularly in T2DM, includes both macrovascular disease as well as the microvascular changes that predispose to diabetic nephropathy, retinopathy, and neuropathy [13], for which there are limited effective medical therapies beyond strict glycemic control, an approach that entails risk of hypoglycemia and perhaps aggravated macrovascular outcomes, as well as blood pressure control.

Addressing this residual cardiovascular risk has advanced considerably. Further lowering of low-density lipoprotein cholesterol (LDL-C), a major component of modifiable ASCVD risk [14], by inhibiting proprotein convertase subtilisin/kexin type 9 (against a background of intense statin therapy) provides incremental reduction in risk [15, 16], with greatest benefit in individuals at highest absolute risk, including those with diabetes or peripheral artery disease [15–18]. Beyond lipids, the CANTOS trial (Canakinumab Antiinflammatory Thrombosis Outcome Study) established that targeting inflammation in patients with high residual risk despite well-controlled LDL-C levels on statin therapy reduces the incidence of major adverse cardiovascular events (MACE) [19], paving the way for exploration of further anti-inflammatory therapies. Additionally, the COMPASS trial (Cardiovascular Outcomes for People using Anticoagulation Strategies) demonstrated that low dose rivaroxaban plus aspirin significantly reduced MACE and major adverse limb events (albeit with a small increase in bleeding events) in patients with stable ASCVD [20].

Together these findings underpin the concept of deploying ‘precision medicine’ to optimize ASCVD prevention. This approach involves focusing on specific modifiable residual cardiovascular risk targets (i.e. lipids, inflammation, or coagulation), according to patient characteristics [21]. Targeting only one component, however, does not eliminate residual cardiovascular risk. The changing landscape of cardiovascular risk drivers provides one explanation, in particular the increasing prevalence of visceral obesity. Visceral obesity and ectopic fat accumulation, particularly in the liver, associate with metabolic diseases and adverse cardiovascular outcomes [5]. Deposition of fat within the liver accompanies a plethora of associated metabolic abnormalities including elevated lipids and blood pressure, insulin resistance, as well as prothrombotic and proinflammatory states [22]. Despite observational association of liver fat content and NAFLD with ASCVD, genetic evidence shows that NAFLD is not causal for ASCVD [23, 24].

Insulin resistant cardiometabolic disease often entails an ‘atherogenic dyslipidemia’, characterized by elevated plasma TG, low HDL-C levels, a preponderance of small, dense LDL particles, and elevated apolipoproteins (apo) B (apoB 100 and apoB48) and C-III concentrations [25, 26]. This dyslipidemic profile is common, especially in low- and middle-income regions where obesity is prevalent, such as Latin America, where it affects nearly 20% of the general adult population [27]. Among high- and very high-risk patients, up to 35% have elevated TG and 10–15% have atherogenic dyslipidemia (Table 1) [26, 28–32]. Atherogenic dyslipidemia therefore offers a particularly attractive target for new therapies to mitigate residual ASCVD risk.

**Table 1:** Prevalence of elevated triglycerides and atherogenic dyslipidemia in the general population and high-risk patient groups

Population	Elevated triglycerides (TG)		Atherogenic dyslipidemia	
	Criterion	Prevalence	Criteria	Prevalence
General populations				
Europe [26]	> 2.2 mmol/L	23.0% (8316/36,160)	TG > 2.2 mmol/L + HDL-C < 1.0 mmol/L (treatment not specified)	6.0% (2169/36,160)
On statin [26]	> 2.2 mmol/L	30.0% (10,848/36,160)		
USA [28, 30]				
Not on statin	≥ 2.26 mmol/L	11.9% (21.5 M/181.0 M)*	TG ≥ 2.26 mmol/L + HDL-C < 1.0 mmol/L (treatment not specified)	6.6% (13.1 M/199.1 M)*
On statin	≥ 2.26 mmol/L	15.4% (6.0 M/38.9 M)*		
High risk populations				
Primary prevention + risk factors [31]	≥ 2.3 mmol/L	20.8% (1591/7641)	Elevated TG + HDL-C < 1.0 mmol/L	9.9% (759/7641)
With T2DM [31]	≥ 2.3 mmol/L	27.5% (562/2046)	Elevated TG + HDL-C < 1.0 mmol/L	14.9% (305/2046)
Clinical ASCVD [29, 32]	> 1.7 mmol/L	34.7% (2938/8467)	TG > 2.0 mmol/L + HDL-C < 1.0 mmol/L in men, < 1.2 mmol/L in women	13–14%**

ASCVD, atherosclerotic cardiovascular disease; HDL-C, high-density lipoprotein cholesterol; M, million; T2DM, type 2 diabetes mellitus; *, projected data; **, Czech component of EUROASPIRE (n = 1484, 1152 men and 332 women)

## Atherogenic dyslipidemia and cardiometabolic risk

Recognition of the contribution of atherogenic dyslipidemia to ASCVD risk is not new [11, 33]. Consistent epidemiological data associate low HDL-C with risk for ASCVD [34]. Genetic studies, however, do not support a protective role of HDL-C in humans [35], and clinical outcomes trials using different therapeutic approaches to target low HDL-C also failed to meet their primary endpoints [36–40]. Together, these findings imply that low HDL-C is a marker of risk and not a therapeutic target.

In contrast to the situation with HDL-C, the case for elevated TG as a biomarker for causal risk has grown in strength [41, 42]. Before discussing the evidence, it merits mention that most of the studies that have evaluated the association between TG and ASCVD risk have measured fasting levels, due to previous concerns that non-fasting samples may overestimate plasma TG. As current evidence does not support this view, either fasting or nonfasting TG concentrations can serve as a marker of increased risk of cardiovascular events and death in both men and women [43–46].

In the PROVE IT-TIMI 22 trial, on-treatment TG < 1.7 mmol/L associated independently with a lower risk of recurrent coronary events in acute coronary syndrome (ACS) patients at LDL-C goal [47]. Pooled analysis of the TNT (Treating to New Targets) and IDEAL (Incremental Decrease in Endpoints Through Aggressive Lipid lowering) trials showed a trend for association between lowering TG levels and reduction in ASCVD events [48, 49]. Elevated TG also predicted recurrent ischemic events in ACS patients treated with statins, as well as progression of coronary atherosclerosis in patients with stable coronary heart disease [50, 51]. Furthermore, long-term (> 20 years) follow-up of the BIP (Bezafibrate Infarction Prevention) Study showed an association between elevated TG and all-cause mortality [52].

In patients with T2DM treated with statin therapy in the ACCORD (Action to Control Cardiovascular Risk in Diabetes) Lipid study, the presence of atherogenic dyslipidemia (TG ≥ 2.3 mmol/L and HDL-C levels ≤ 0.88 mmol/L) associated with an increase in cardiovascular event rates [53]. These findings derive support from real-world data in statin-treated diabetes patients with elevated TG (2.3–5.6 mmol/L), which showed higher rates for non-fatal myocardial infarction (MI, by 30%) compared with patients with lower TG [54]. This result undoubtedly translates to greater healthcare costs associated with management of these complications [55]. Furthermore, as previously noted, high TG and low HDL-C associate with diabetic microangiopathy, in particular nephropathy, as supported mainly by evidence from observational studies, especially in individuals with LDL-C at goal [13].

## Which is the risk factor: triglycerides or triglyceride-rich lipoproteins?

**Fig. 1:**
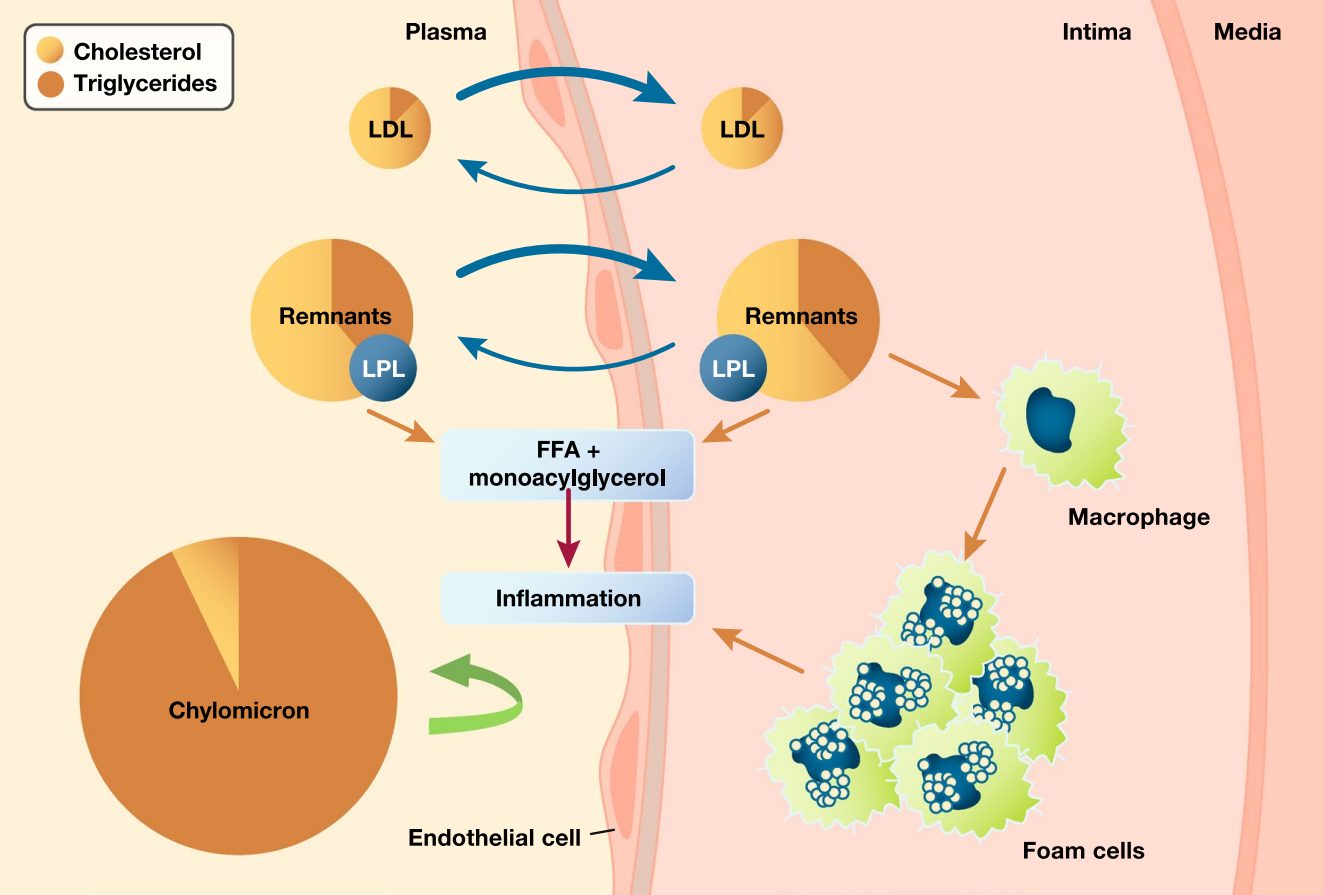
Remnant lipoproteins accumulate in the arterial wall where they elicit inflammation. This provides a mechanistic basis for a causal role in atherosclerosis. Adapted from Nordestgaard and Varbo [56] with permission. FFA, free fatty acids; LDL, low-density lipoproteins; LPL, lipoprotein lipase

The atherogenic entities of particular interest are, however, TG-rich lipoproteins and their remnants, for which circulating TG levels serve as a biomarker. TG-rich lipoproteins encompass a mixture of chylomicrons (synthesized in the intestine) and very low-density lipoprotein (VLDL) particles (synthesized in the liver) (Fig. 1) [56]. Under fasting conditions, the liver secretes both VLDL1 and VLDL2 containing apo B100; the larger form, VLDL1, carries most of the TG and associates with NAFLD [57]. Lipoprotein lipase (LpL) subsequently hydrolyzes both VLDL classes to form smaller and denser lipoprotein particles. The action of LpL on VLDL can generate endogenous, natural PPARα ligands, resulting in anti-inflammatory and anti-atherosclerotic responses [58]. Consistent with this finding, overexpression of LpL in settings where it is not usually found, e.g. cardiac myocytes, induces expression of PPARα target genes [59, 60]. In the post-prandial phase, the intestine secretes chylomicrons containing apo B48, which subsequently undergo hydrolysis by LpL, with release of free fatty acids and formation of chylomicron remnants. LpL activity undergoes both pre- and post-transcriptional regulation mediated by free fatty acids, apo C-II, apo C-III, apo A-V, angiopoietin-like members 3, 4, 8 (ANGPTL 3, 4, 8), and glycosylphosphatidylinositol anchored HDL binding protein 1 (GPIHBP1) [56, 61, 62].

Insulin resistance drives dysregulation of the metabolism of TG-rich lipoproteins by two mechanisms. On the one hand, excess flow of free fatty acids to the liver, compensatory hyperinsulinemia, together with concomitant activation of enzymes involved in hepatic de novo lipogenesis (DNL) contribute to overproduction of VLDL1 particles. DNL generates malonyl-CoA that inhibits carnitine palmitoyl transferase I, resulting in reduced uptake of long-chain fatty acyl groups into mitochondria and hence reduced beta-oxidation. On the other hand, increased secretion of apo C-III mediates impaired clearance of VLDL1-TG. The combination of hepatic TG-rich lipoprotein overproduction and inefficient clearance increases the residence time of circulating TG-rich lipoproteins [63]. This delayed clearance enhances the exchange of components such as cholesteryl ester, TG, and apolipoproteins between lipoproteins, and further remodeling by hepatic lipase results in cholesterol-enriched remnants, small dense LDL particles and low plasma HDL-C levels [63].

## Triglyceride-rich lipoproteins, remnants and ASCVD

TG-rich lipoproteins and their remnants contain both TG and cholesterol. As all cells in the body readily degrade TG, the enhanced ASCVD risk likely results from the cholesterol component of TG-rich lipoproteins and their remnants (referred to as ‘remnant cholesterol’ and estimated in clinical practice as total cholesterol − [LDL-C + HDL-C]). Indeed, with the exception of very large particles such as chylomicrons, these lipoproteins and their remnants can enter the arterial wall, ultimately depositing their cholesterol load in the atherosclerotic plaque (Fig. 1) [56, 64, 65]. Post hoc analysis of the TNT study also showed that TG-rich lipoprotein cholesterol concentration was an independent marker of residual ASCVD risk [66].

### Insights from Mendelian randomization and genetic studies

Mendelian randomization studies strongly support the causality of remnant cholesterol carried by TG-rich lipoproteins in ASCVD. Elevated levels of remnant cholesterol associate with both increased observational and genetic risk for ASCVD, independent of HDL-C levels [67, 68]. Furthermore, while elevated nonfasting remnant and LDL-C levels each associate with increased risk of ischemic heart disease and MI, only elevated remnant cholesterol concentration associates with increased risk of all-cause mortality [69]. Elevated nonfasting remnant cholesterol may also contribute to the residual risk of all-cause mortality in individuals with established ischemic heart disease [70]. These findings reinforce the long-held view that postprandial lipemia contributes to atherogenesis, as during an ordinary day, individuals spend more time in the nonfasting than fasting state [71, 72]. Mechanistically, the atherogenicity of elevated remnant cholesterol may involve inflammation [73], as the Copenhagen studies show that elevated plasma C-reactive protein levels (> 2 mg/dL), a marker of inflammation, commonly accompany elevated TG levels (≥ 1.7 mmol/L) [74].

**Fig. 2:**
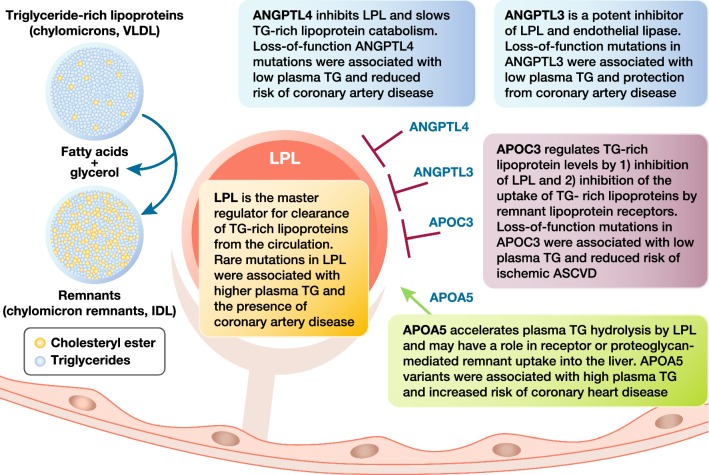
Genetic studies suggest novel approaches for the management of hypertriglyceridemia focused on key targets involved in the regulation of triglyceride-rich lipoprotein metabolism: apolipoprotein C-III (encoded by *APOC3*), angiopoietin-like proteins (ANGPTL) 3 and 4, apolipoprotein A V (apo A V) and lipoprotein lipase (LPL)0 [75–83]. IDL, intermediate-density lipoproteins; TG, triglycerides; VLDL, very low-density lipoproteins

Genetic studies which investigated the impact of mutations in genes involved in TG-rich lipoprotein metabolism have strengthened evidence for a link between TG-rich lipoproteins, their remnants and ASCVD risk (Fig. 2**)**. Loss-of-function (LOF) variants in genes encoding apo AV and LpL associate with lifelong higher plasma TG levels and an increased risk of coronary artery disease [75–78], whereas LOF mutations in *APOC3* and *ANGPTL4* associate with lifelong decreased plasma TG levels and reduction in the risk of coronary artery disease [79–82]. These data are highly consistent with the action of LpL releasing endogenous PPARα ligands that limit atherosclerosis [60]. Evidence also implicates ANGPTL3 in control of TG and promotion of coronary risk [83]. Thus, mutations in all five genes that regulate TG-rich lipoprotein metabolism impact the subsequent risk for ASCVD.

The potential of apo C-III as a therapeutic target merits emphasis. Clinical evidence has already established apo C-III as a cardiovascular risk predictor independent of TG levels [84]. Accumulating preclinical studies also suggest that apo C-III exerts lipid-independent pro-inflammatory effects [85]. Individuals with diabetes mellitus have elevated apo C-III concentrations, in part mediated via effects on the functionality of the β-cell, affecting intracellular calcium handling and insulin sensitivity [86]. Therefore, targeting apo C-III may offer benefits beyond TG lowering in patients with diabetes.

### Insights from trials of TG-lowering therapies

Guidelines recommend fibrates (PPARα agonists) and omega-3 fatty acids for the management of hypertriglyceridemia, usually as an add-on to primary statin treatment [6, 87–89]. Cardiovascular outcomes studies with these agents have, however, yielded mixed results. In the case of the major fibrate trials, none recruited selectively patients with high TG levels. For example, the ACCORD Lipid study, which aimed to examine the benefit of adding a fibrate to statin therapy in patients with T2DM, had no TG entry criteria, and the median TG was only 1.8 mmol/L (interquartile range 1.3 to 2.6 mmol/L). The study showed no significant benefit of add-on fenofibrate treatment on residual cardiovascular risk [53]. Despite these shortcomings, post hoc analyses of the major fibrate trials did indicate benefit in individuals with atherogenic dyslipidemia [90]. Moreover, long-term follow-up of patients in ACCORD Lipid showed continued benefit from fenofibrate in this subgroup [91]. Reinforcing the relevance of elevated TG-rich lipoproteins to ASCVD risk, regression analysis including data from the major fibrate trials showed a 54% (95% confidence interval 5 to 78%) reduction in cardiovascular events per 1 mmol/L reduction in TG levels [56]. As with any treatment, however, these agents have limitations, predominantly due to drug–drug interactions (in particular, between gemfibrozil and statins), or effects on renal function (notably with fenofibrate, reversible elevation in serum creatinine), or hepatic safety [7–10].

Whether omega-3 fatty acids reduce cardiovascular events has engendered debate. While the JELIS (Japan EPA Lipid Intervention Study) trial reported a 19% reduction in major coronary events [92], other studies were inconclusive, perhaps because they used lower doses of omega-3 fatty acids than required clinically to lower TG substantially [93]. Recently, however, REDUCE–IT (Reduction of Cardiovascular Events with Icosapent Ethyl–Intervention Trial) showed that treatment with high dose (4 g) eicosapentaenoic acid ethyl ester in high-risk individuals (58% with diabetes) with elevated TG (median 2.4 mmol/L [interquartile range 2.0–3.1 mmol/L]) resulted in relative reductions of 25% in the incidence of MACE and 20% in cardiovascular mortality against a background of well-controlled LDL-C levels on statin treatment [94]. Although the cardiovascular outcomes benefit exceeded that anticipated by the magnitude of TG lowering (18.3%), suggesting the involvement of other mechanism(s), selection of an appropriate patient population, including both primary (30%) and secondary prevention groups with elevated TG, supports REDUCE-IT as a landmark trial supporting the concept of targeting elevated TG to reduce residual ASCVD risk. The question is, can application of a precision medicine approach to improve the clinical profile of fibrates (PPARα agents), also offer potential to mitigate residual ASCVD risk?

## PPARα: the nuclear receptor ‘hub’ for TG-rich lipoprotein metabolism

**Fig. 3:**
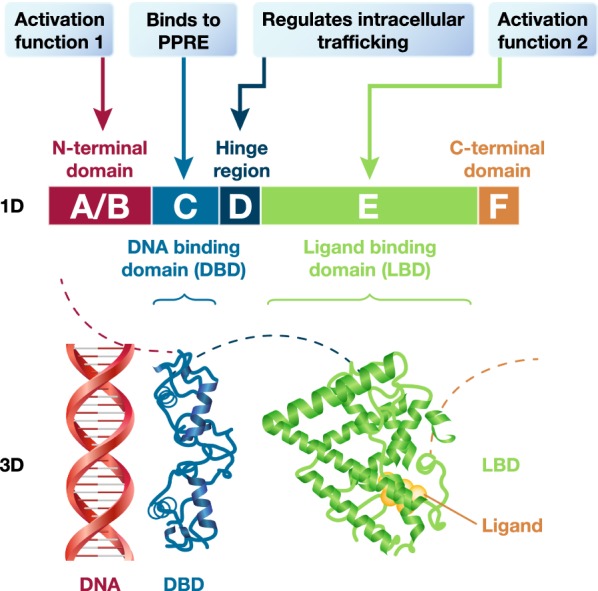
Structural organization of nuclear receptors. The ligand binding domain of PPARα includes the ligand dependent activation function 2 interface. PPRE, peroxisome proliferator response element

Understanding the role of PPARα in lipid metabolism is fundamental to defining the SPPARMα concept. PPAR belongs to the extended family of nuclear receptors, ligand-dependent transcriptional regulators—‘hubs’—that control key metabolic processes involved in development, reproduction, metabolism, and inflammation. The PPAR subfamily of nuclear receptors comprises three isotypes: PPARα, PPARβ/δ and PPARγ, each encoded by separate genes and with a unique albeit overlapping tissue distribution. These three isotypes share a common structural organization, namely, a variable N-terminal domain with a ligand-independent activation function, a conserved DNA binding domain, and a C-terminal ligand-binding domain, which contains the ligand-dependent activation function 2 (AF2) (Fig. 3) [95]. Attention has focused on PPARα given that (1) it is highly prevalent in metabolically active tissues such as the liver, kidney, heart, muscle, brown adipose, and macrophages, and (2) has a key role in transcriptional regulation of lipoprotein metabolism, specifically fatty acid transport and beta-oxidation, as well as vascular inflammation [95]. Hepatic PPARα agonism accounts for most of these effects. Under circumstances of diminished hepatic PPARα function, PPARα-dependent regulation of fatty acid oxidation in peripheral tissues may also become relevant [96].

**Fig. 4:**
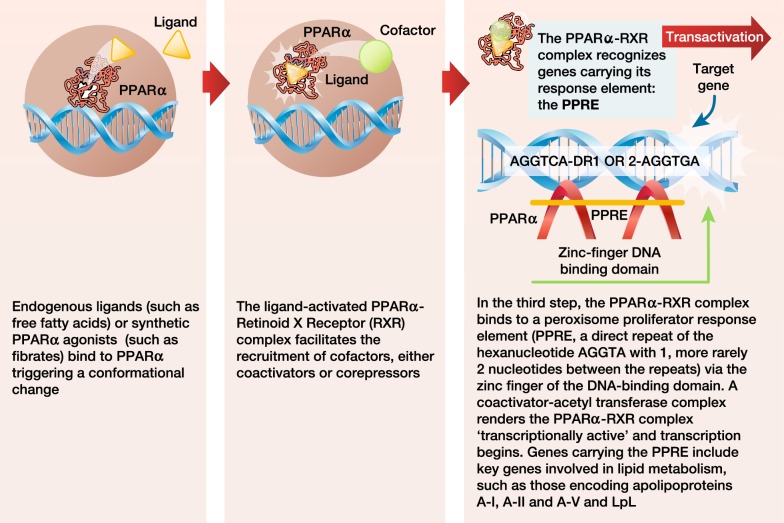
Transcriptional activation of PPARα is a three-part process

The ligand binding domain, which accommodates the lipophilic ligands and also harbors a transcriptional activation function at the C-terminus, has a critical role [97]. Binding of an agonist to the ligand binding domain triggers a conformational change. The activated nuclear receptor then binds to a specific DNA sequence in the promoter region of the target gene, resulting in activation of gene transcription (a process referred to as transactivation). The nuclear receptor may also bind to a repressor protein that prevents transcription of other genes (referred to as transrepression) [98]. For PPARα, transcriptional activation is a three-step process (Fig. 4) [95, 99]. Binding of an endogenous ligand (e.g. prostaglandins, leukotrienes, and medium-long-chain free fatty acids, especially when released by LpL) or a synthetic PPARα agonist (e.g. a fibrate) to PPARα triggers a conformational change which stabilizes the ligand binding domain and facilitates the recruitment of a specific profile of coactivators and/or the release of corepressors [100]. Of the 320 known cofactors that bind to nuclear receptors, 38 bind to PPAR. Such PPAR cofactors include PGC-1α (peroxisome proliferator-activated receptor-γ coactivator-1α), SRC1 (steroid receptor coactivator 1), and NcoR1 (nuclear receptor co-repressor 1). The ligand-activated PPARα forms a heterodimeric complex with another ligand-activated nuclear receptor, the Retinoid X Receptor (RXR), and binds to a specific DNA sequence in the promotor region of target genes referred to as a peroxisome proliferator response element (PPRE) [101]. Activation by a coactivator-acetyl transferase complex results in the expression of key genes involved in lipid metabolism, including those encoding apo A-I, A-II, A-V and C-III, LpL, scavenger receptor BI, adenosine triphosphate-binding cassette transporter A1 (ABCA1), ATP binding cassette subfamily G member 1 (ABCG1), and acyl CoA synthase. Thus, the net effects of PPARα activation on lipid metabolism include increases in HDL production, VLDL clearance, and LDL particle size, with downstream decreases in VLDL production, and LDL particle concentration [95, 102]. PPARα can also compete for co-activators of the cytokine-activated nuclear factor-κB, inhibiting the expression of pro-inflammatory genes, resulting in reduced vascular inflammation [95]. Indeed, studies demonstrated the anti-inflammatory activity of PPARα more than 20 years ago [103–106]. More recent findings have shown that PPARα activation in mouse liver reduces the CCAAT/enhancer binding protein (C/EBPβ), as well as nuclear factor-κB protein expression, resulting in lower levels of C-reactive protein, interleukin-6 and prostaglandins [107].

PPARα may also regulate glucose homeostasis and reduce thrombogenesis [95, 108]. Activation of PPARα may shift the balance of glucose versus fatty acid as the major energy source for intracellular metabolism. In the metabolically challenged liver in a glucose-rich environment, suppression of the tricarboxylic acid cycle in the mitochondria leads to an increase in acetyl co-A levels in the cytoplasm, impairing cellular homeostasis (for example, a decrease in transcription of autophagy-related genes, and an increase in oxidative stress) [109]. Similar phenomena may occur in activated macrophages, cells which contribute to the pathogenesis of ASCVD [110]. Instead, PPARα activation may promote beta oxidation, and the tricarboxylic acid cycle, triggering starvation signaling-like responses and ameliorating intracellular dysmetabolism. Taken together, these findings suggest that PPARα has the potential for addressing multiple contributors to residual cardiovascular risk.

**Table:** 

In summary, PPARα is the nuclear receptor ‘hub’ for transcriptional regulation of lipoprotein metabolism and vascular inflammation. Conformational changes induced by binding of a ligand (either endogenous or synthetic) to PPARα facilitate the recruitment of a specific profile of cofactors, which either promote or repress expression of target genes involved in key metabolic pathways.	

## Defining the SPPARMα concept

Development of selective estrogen receptor modulators (SERMs) provides an analogy for the SPPARMα concept. Depending on the tissue, SERMs can act as either agonists or antagonists of the estrogen receptor, with the cofactor milieu and structure of the bound receptor-ligand complex influencing tissue-specific cellular transcriptional activity and the subsequent profile of physiological effects [111]. Modulation of the estrogen receptor activity of the ligand permitted promotion of specific beneficial effects (in breast tissue) and avoidance of adverse effects (such as uterotropic effects) [112]. SERMs therefore suggest a ‘blueprint’ for modulating the ligand binding profile of PPARα, to improve potency and selectivity and potentially, limit tolerability issues seen with fibrates. This rationale underpins the SPPARMα concept [113].

The PPARα receptor has a large ligand binding pocket which can bind a range of endogenous and synthetic ligands, each capable of triggering specific conformational changes, resulting in a characteristic cofactor binding pattern. Different transcriptional responses seen between endogenous LpL-released fatty acids, prescription omega 3 fatty acids and different pharmacologic forms of fibrates are strongly supportive of the SPPARMα concept [60]. Modulation of the receptor–cofactor binding profile of the PPARα ligand tuned tissue- and gene-selective effects and, thus physiological responses [113]. LY-518674 was among the first SPPARMα agonists evaluated. Its higher potency than fenofibrate in vitro did not translate to superior efficacy in lowering TG and raising HDL-C in patients with atherogenic dyslipidemia. Additionally, there were safety concerns, notably an increase in serum creatinine (similar to that observed with fenofibrate) in clinical studies [114]. A subsequent search for a novel SPPARMα involved the synthesis and screening of over 1300 compounds before identification of one compound, K-877 (subsequently named pemafibrate), with potential SPPARMα activity.

**Table:** 

In summary, binding interactions between the ligand and the PPARα receptor modulate the receptor–cofactor binding profile; this rationale underpins the SPPARMα concept.	

## Differentiating SPPARMα and PPARα agonists: pharmacology

**Fig. 5:**
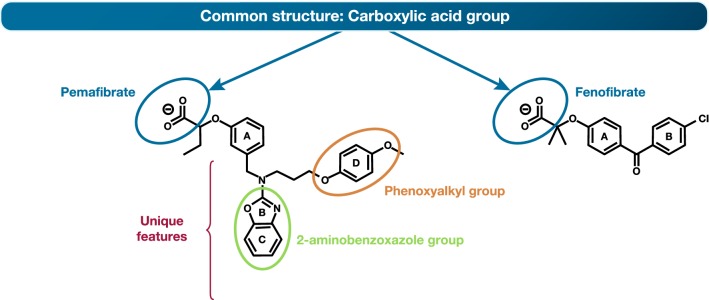
Structures of a SPPARMα (pemafibrate) and PPARα (fenofibrate) showing shared and unique regions. This Y-structure of pemafibrate results in improved fit with the PPARα ligand binding site compared with fenofibrate (see Additional files 1, 2)

As in the case of SERMs, structural features allow differentiation of this SPPARMα agonist from PPARα agonists [115]. Specifically, the addition of unique benzoxazole and phenoxyalkyl side-chains confer a Y-shape to the SPPARMα agonist pemafibrate, contrasting with the linear structure of PPARα agonists such as fenofibrate (Fig. 5). In silico computer simulation, which enables coupling of information relating to structure and sequence, demonstrated that this SPPARMα agonist binds to the entire Y-shaped ligand binding pocket with an enhanced induced fit compared with PPARα agonists such as fenofibrate (Fig. 5, Additional files 1, 2). Changes in PPARα conformation form a new interface which binds to PGC-1α, a transcriptional coactivator, resulting in complete activation of PPARα [116]. Quantitative evaluation of ligand docking using computer-linked fragment molecular orbit analysis predicted which amino acids mediate binding to the SPPARMα agonist, as confirmed by mutation experiments. Identification of the key role of PGC-1α in binding is important, given that it regulates metabolic adaptation, and thus influences the development of systemic insulin resistance, glucose intolerance and insulin deficiency [117]. This SPPARMα agonist exhibited greatly enhanced PPARα potency and selectivity in cell-based transactivation assays, > 2500-fold more potent than fenofibric acid, the active metabolite of fenofibrate, and > 5000-fold more specific for human PPARα than either PPARγ or δ [118].

Transcriptome analysis showed that while 11 of the main 20 genes induced by pemafibrate or fenofibrate participate in carbohydrate and lipid metabolism, there were differences in the magnitude of effect. For example, in human hepatocytes this SPPARMα agonist further induced key target genes such as *VLDLR* and *ABCA1* at 10-fold lower concentration than fenofibrate (10 μM vs. 100 μM) [119]. SPPARMα agonism predominantly induced mitochondrial genes encoding 3-hydroxy-3-methylglutaryl-CoA (HMG-CoA) synthase 2, fatty acid-binding protein 1 (FABP1), and pyruvate dehydrogenase kinase isozyme 4 (PDK4), involved in maintaining glucose homeostasis and increasing ketone body utilization. This SPPARMα agonist (but not fenofibric acid) also augmented the expression of fibroblast growth factor 21 (FGF21) [119], a metabolic regulator with favourable effects on glucose and lipid metabolism [120]. Experimentally, FGF21 induces fatty acid oxidation, ketogenesis and gluconeogenesis, as well as suppresses lipogenesis; [121] some reports have also shown this effect with fibrates [122]. In addition, there was increased expression of genes involved in the regulation of the innate immune system (mannose-binding lectin 2 [MBL2]), inflammation, blood pressure (glutamyl aminopeptidase [ENPEP]), and glucose and energy homeostasis, implying the potential for effects beyond lipid modification [119]. Moreover, this SPPARMα agonist had no effect on peroxisome biogenesis genes in human hepatocytes, suggesting that it does not stimulate peroxisome proliferation, and thus avoids hepatic adverse effects in humans [119].

## SPPARMα in pre-clinical studies

**Fig. 6:**
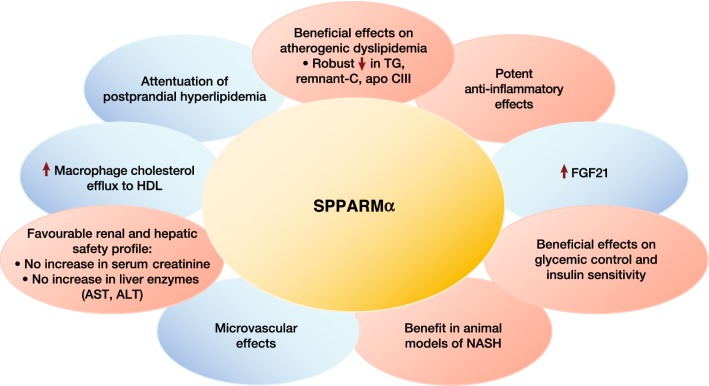
Differentiation of the pharmacological profile of a SPPARMα (pemafibrate) based on available data. ALT, alanine aminotransferase; apo apolipoprotein; AST, aspartate aminotransferase; C, cholesterol; FGF21, fibroblast growth factor 21; HDL, high-density lipoprotein; TG, triglycerides

Multiple preclinical studies investigated the pharmacological profile of this novel SPPARMα agonist (reviewed in reference 118 and summarized in Fig. 6). Compared with fenofibrate, pemafibrate resulted in greater TG-lowering and elevation in HDL-C in animals with hypertriglyceridemia [118, 123], and in C57BL/6J mice fed a high-fat diet, attenuated postprandial hypertriglyceridemia more effectively, by suppressing the postprandial increase in chylomicrons and accumulation of chylomicron remnants [124]. This SPPARMα agonist produced similar lipid modulating actions in the liver and intestine [125].

Beyond lipid effects, this SPPARMα agonist also promoted potent anti-inflammatory effects, increased macrophage cholesterol efflux to HDL, inhibited lipid deposition in the aorta, and attenuated atherosclerotic lesion development in animals [126, 127]. Evidence from obese mice also suggests that this SPPARMα agonist ameliorates visceral obesity-induced hyperglycemia and elevated TG-rich lipoproteins, possibly mediated by an increase in circulating FGF21 levels, as well as enhanced expression of genes involved in thermogenesis and fatty acid oxidation in both white and brown adipose tissue [128]. In rodents with non-alcoholic steatohepatitis (NASH), pemafibrate improved liver dysfunction by modulation of hepatic lipid turnover and energy metabolism [129]. Finally, this SPPARMα agonist may produce beneficial microvascular benefits, with evidence of reduction of diabetic nephropathy in diabetic *db/db* mice, attributed, at least partly, to inhibition of renal lipid content and oxidative stress [130].

**Table:** 

In summary, preclinical studies have revealed that enhanced potency, selectivity and cofactor binding profile differentiate this novel SPPARMα agent from traditional non-selective PPARα agonists. Clinically relevant genes regulated by this SPPARMα agonist include those involved in regulation of lipoprotein metabolism, such as *VLDLR* and *ABCA1,* inflammation, the innate immune system (*MBL2*) and energy metabolism (*FGF21*). In preclinical studies, this SPPARMα activator had lipid modifying and anti-inflammatory effects, as well as regulatory effects in glucose homeostasis and liver dysfunction.	

## Differentiating SPPARMα and PPARα agonists: clinical trial evidence

### Efficacy

Thus, the pharmacological profile of this SPPARMα agonist suggests benefit in the management of atherogenic dyslipidemia, in particular elevated TG-rich lipoproteins and remnant cholesterol common in overweight patients with T2DM [131]. A phase II dose-ranging trial (oral pemafibrate 0.025–0.2 mg twice daily) in Japanese patients with elevated TG (≥ 2.3 mmol/L) and low HDL-C (< 1.3 mmol/L in men and < 1.4 mmol/L in women) defined the clinically relevant dose range for this SPPARMα agonist [132]. After 12 weeks, this agent produced dose-related reductions from baseline in TG (by 30.9% to 42.7%), VLDL-cholesterol (by 24.3% to 48.4%), remnant-cholesterol (by 32.3% to 50.1%), apo B48 (by 28.4% to 55.9%), and apo C-III (by 2.2% to 34.6%), as well as an increase in HDL-C (by 11.9% to 21.0%), compared with both placebo and micronized fenofibrate 100 mg once daily, with maximal effects at a dose of 0.2–0.4 mg daily (Table 2). Treatment with this SPPARMα agent also ameliorated the atherogenic lipoprotein profile, reducing the proportion of small and very small LDL particles, and increasing small and very small HDL particles. Reduction in non-HDL-C and apo B100 was less pronounced (~ 10%) during pemafibrate therapy [132]. In another study in Japanese patients with high TG and low HDL–C, pemafibrate 0.2 mg or 0.4 mg daily was significantly more effective than a low dose of fenofibrate (solid dispersion tablet 106.6 mg, equivalent to micronized fenofibrate 134 mg daily) and non-inferior to fenofibrate 200 mg daily [133]. Subsequent phase II/III trials in Japanese and European patients with elevated TG with or without T2DM confirmed the lipid-modifying activity of this SPPARMα agonist, in particular robust and sustained lowering of remnant cholesterol (by up to 80%), and TG and apo C-III (by ~ 50%) [134–139]. As this SPPARMα agent depends predominantly on excretion by the liver [140], the TG-lowering response with pemafibrate does not vary with baseline estimated glomerular filtration rate (eGFR) [141]. Table 2 summarizes clinical trials with this SPPARMα agonist.

**Table 2:** Overview of published Phase II/III clinical trials with pemafibrate

Citation	Patients	Treatment daily dose (mg) and duration	Key findings
Ishibashi [132] Phase II	N = 224 with high TG + low HDL-C^a^	Pemafibrate 0.05, 0.1, 0.2, 0.4 Fenofibrate 100 Placebo 12 weeks	LS mean [SE] percent changes from baseline to 12 weeks (pemafibrate 0.4 vs. fenofibrate) Decrease in TG: 42.7 [6.7]% vs. 29.7 [6.7]% Increase in HDL-C: 21.0 [2.8]% vs. 14.3 [2.8]% LS mean [SD] percent decrease (pemafibrate 0.4 vs. fenofibrate) VLDL-C: 48.4 [27.5]% vs. 25.8 [29.7]%** Remnant-C: 50.1 [31.8]% vs. 31.8 [35.0]% Apo C-III: 33.4 [19.2]% vs. 27.2 [18.9]% Increase in FGF21 (pemafibrate vs. fenofibrate)*** The incidence of adverse events with pemafibrate, fenofibrate or placebo was similar Conclusion: In dyslipidemic patients with high TG and low HDL–C, pemafibrate improved TG, HDL-C, and other lipid parameters without increasing adverse events, compared to placebo and fenofibrate
Ishibashi [134] Phase III	N = 225 with high TG and low HDL-C^b^	Pemafibrate 0.2, 0.4 vs. Fenofibrate 106.6 24 weeks	LS mean [SE] reduction from baseline to 24 weeks in TG: 46.2 [2.0]% with pemafibrate 0.2 and 45.9 [1.9]% with 0.4 vs. 39.7 [1.9]% with fenofibrate* At 24 weeks, significant ↓ALT** and GGT** with pemafibrate compared with fenofibrate Conclusion: Pemafibrate was superior to fenofibrate in terms of serum TG-lowering effect and hepatic and renal safety
Arai [133] Phase III	N = 526 with high TG and low HDL-C^a^	Pemafibrate 0.1, 0.2, 0.4 Fenofibrate 100, 200 vs. placebo 12 weeks	Non-inferior LS mean [SE] decrease in TG vs. fenofibrate 200: 46.7 [1.6]% with pemafibrate 0.2 and 51.8 [2.0]% with 0.4 vs. 51.5 [1.6]% No dose-dependent increase in adverse events with pemafibrate The incidence of adverse events for all pemafibrate doses was similar to that for placebo and fenofibrate 100 and significantly lower than fenofibrate 200 mg* Conclusion: The favorable safety profile of pemafibrate, with fewer adverse effects on kidney/liver-related tests and fewer adverse events over fenofibrate 200 mg/day, may justify the use of this novel and potent treatment option for reducing TG in a broader range of patients
Arai [135] Phase III	2 trials, dyslipidemia on statin therapy Trial A^c^: N = 188 Trial B^d^: N = 423	Trial A Pemafibrate 0.1, 0.2, 0.4 vs. placebo 12 weeks Trial B Pemafibrate: 0.2, 0.2/0.4^g^ vs. placebo 24 weeks	Trial A LS mean [SE] decrease in TG at 12 weeks: 53.4 [3.8]% with pemafibrate 0.2, 52.0 [3.9]% with 0.4 vs. 6.9 [4.0]% with placebo, p < 0.001 Trial B LS mean [SE] decrease in TG at 24 weeks: 46.8 [2.6]% with pemafibrate 0.2, 50.8 [2.5]% with 0.2/0.4 vs. 0.8 [3.0]% with placebo, p < 0.001 34% of patients were titrated to the higher dose In both trials, pemafibrate ameliorated the atherogenic lipoprotein profiles, i.e. ↓small LDL, ↑ larger LDL and ↓larger HDL, ↑ small HDL Conclusion: These results strongly support the favourable benefit-to-risk ratio of pemafibrate add-on therapy in combination with statin treatment
Araki [136] Phase III	N = 166, T2DM with high TG^e^	Pemafibrate 0.2, 0.4 vs. placebo 24 weeks	LS mean reductions with pemafibrate vs. placebo TG: 44.3% with 0.2, 45.1% with 0.4 vs. 10.8%, p < 0.001 Non-HDL-C 6.3% and 12.5%, remnant-C 45.7% and 49.2%, apo B100 9.1% with 0.2 mg, apo B48 43.7% and 50.6%, and apo C-III 32.5% and 34.0%, all p < 0.001 HOMA-insulin resistance score with 0.2 mg, p < 0.05 Both pemafibrate doses significantly ↑ FGF21, p < 0.001 Conclusion: Pemafibrate significantly ameliorated lipid abnormalities and was well tolerated in patients with T2DM with hypertriglyceridemia
Yamashita [137]	N = 33 with atherogenic dyslipidemia^f^	Crossover study, pemafibrate 0.4 or placebo Each period was 4 weeks	Significant (p < 0.001) mean percent LS [SE] changes with pemafibrate vs. placebo Decreases in TG (39.8 [19.4]% vs. increase of 22.5 [36.0]%), non-HDL-C (12.0 [19.9]% vs. increase of 3.5 [12.6]), remnant-C (50.6 [24.5]%), vs. increase of 17.5 [35.6]%, and apo C-III (31.3 [20.1]% vs. increase of 11.6 [28.3]%) Increases in HDL-C (16.1 [15.0]% vs. decrease 1.4 [10.6]%), apo A–I (8.3 [9.1]% vs. 1.3 [9.8]%) and apo A-II (38.2 [17.4]% vs. 5.5 [12.6]%) Pemafibrate significantly increased FGF21 (p < 0.001), and decreased hsCRP and serum amyloid A (p < 0.01) vs. baseline Pemafibrate improved postprandial hyperlipidemia Pemafibrate improved HDL quality (macrophage cholesterol efflux capacity) and increased preβ1 HDL and HDL3 Conclusion: Pemafibrate enhances reverse cholesterol transport and may retard the progression and even promote the regression of atherosclerosis by comprehensively ameliorating the atherogenic lipid profile

ALT, alanine aminotransferase; FGF21, fibroblast growth factor 21; GGT, gamma glutamyl transferase; HDL-C, high-density lipoprotein cholesterol; hsCRP, high-sensitivity C-reactive protein; LS, least squares; SD, standard deviation; SE, standard error; VLDL-C, very low-density lipoprotein cholesterol; TG, triglycerides

* p < 0.05, ** p < 0.01, *** p < 0.001 vs. fenofibrate

Dyslipidemia defined as:

^a^TG ≥ 2.23 mmol/L and HDL-C < 1.3 mmol/L in men < 1.4 mmol/L in women

^b^TG ≥ 1.7 mmol/L and < 5.7 mmol/L and HDL-C < 1.3 mmol/L in men and < 1.4 mmol/L in women

^c^TG ≥ 2.23 mmol/L and non-HDL-C ≥ 3.9 mmol/L

^d^TG ≥ 2.23 mmol/L

^e^TG ≥ 1.7 mmol/L

^f^TG ≥ 1.7 mmol/L and < 4.5 mmol/L and HDL-C < 1.3 mmol/L in men and < 1.4 mmoL/L in women

^g^Pemafibrate was up titrated from 0.2 mg/day to 0.4 mg/day after week 12 if fasting TG were ≥ 1.7 mmol/L at week 8

Subsequent studies showed that treatment with this SPPARMα agonist significantly reduced the postprandial area under the curve for TG, apoB 48, and remnant cholesterol for patients with and without T2DM [136, 139]. In patients with atherogenic dyslipidemia, treatment with pemafibrate not only significantly increased HDL-C, apo A-I, and apo A-II levels, but also improved indices related to HDL function, as shown by increases in prebeta-HDL, smaller HDL particles (HDL3 and HDL2), and macrophage cholesterol efflux capacity, a marker of the ability of HDL to mediate reverse cholesterol transport [139]. Some evidence also suggested non-lipid effects with pemafibrate 0.2 to 0.4 mg daily, including beneficial effects on glycemic control and insulin sensitivity in patients with and without T2DM [132, 136, 142]. In a hyperinsulinemic–euglycemic clamp study in patients with elevated TG (mean 3.3 mmol/L [standard deviation 1.10 mmol/L]) and insulin resistance, pemafibrate 0.4 mg daily for 12 weeks significantly increased splanchnic glucose uptake, although there was no change in peripheral glucose uptake rates compared with placebo [143]. Treatment with pemafibrate also significantly increased FGF21 to a greater extent than 100 mg micronized fenofibrate [132, 134–136, 139], and lowered biomarkers of inflammation (C-reactive protein and serum amyloid A) [139].

### Safety

As with all novel therapies, clinicians and patients alike share concerns regarding benefits versus risks. Across all trials, this SPPARMα agonist was generally well tolerated, particularly with respect to renal and hepatic safety signals. The incidence of adverse events with pemafibrate resembled that of placebo (or statin alone in pemafibrate combination treatment trials) and showed no association with pemafibrate dose. Moreover, there were fewer adverse effects relating to renal or hepatic function with this SPPARMα agonist than with fenofibrate 200 mg daily [118, 142]. Pooled analyses of phase II/III studies showed significant improvement in liver function tests (alanine aminotransferase, gamma glutamyl transferase, and bilirubin) with this SPPARMα agonist administered over 12–24 weeks [143]. Importantly, and in contrast to studies with fenofibrate which showed reversible increases in serum creatinine and a decline in eGFR [8, 9], no pemafibrate dose studied elevated serum creatinine over up to 52 weeks in patients with or without pre-existing renal dysfunction [142]. In addition, while both pemafibrate and fenofibrate (solid-dispersion tablet 106.6 mg daily) increased serum homocysteine, the effect was less with pemafibrate [134].

In summary, the sum of evidence from clinical studies provides further support for the SPPARMα concept. Briefly, treatment with this SPPARMα agonist resulted in robust and sustained lowering of TG-rich lipoproteins, remnant cholesterol, and apo C-III, together with improvement in the atherogenic lipoprotein profile, as well as attenuation of postprandial hyperlipidemia in patients with and without T2DM. Pemafibrate also favourably affected glycemia, FGF21, and inflammatory markers. The safety data for this SPPARMα agonist are encouraging over the relatively short duration of exposure in clinical trials so far, especially for renal and hepatic safety, with no evidence of elevation in serum creatinine during treatment. There remain, however, a number of outstanding questions. Chief among them is whether translation of the SPPARMα concept to the clinic will reduce residual cardiovascular risk and prove safe during long-term treatment.

**Table:** 

Clinical trials support the SPPARMα concept, showing robust and sustained reduction of TG-rich lipoproteins in patients with atherogenic dyslipidemia, with or without T2DM. The risk versus benefit profile so far is also encouraging, especially the lack of any effect on serum creatinine during treatment, although longer-term safety data are needed.	

## Unanswered questions: SPPARMα, residual vascular risk and NAFLD

A number of lines of evidence suggest that treatment with this SPPARMα agonist could limit atherosclerotic lesion progression. In preclinical studies, pemafibrate promoted macrophage cholesterol efflux to HDL and attenuated atherosclerotic lesion development [126, 127] and, in patients with atherogenic dyslipidemia, pemafibrate treatment improved macrophage cholesterol efflux capacity [139]. Moreover, this SPPARMα agent robustly reduces TG, and exerts potent anti-inflammatory effects. Therefore, pemafibrate may offer a novel approach to target residual cardiovascular risk in high-risk patients with atherogenic dyslipidemia, especially those with T2DM. The data so far support testing the SPPARMα concept to determine whether therapeutic lowering of TG-rich lipoproteins with pemafibrate, on a background of best evidence-based treatment including statin therapy, will reduce incident ASCVD events and exhibit long-term safety.

**Fig. 7:**
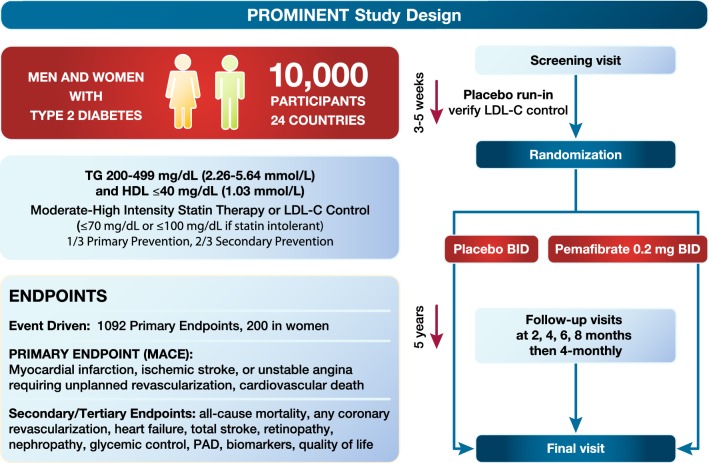
Design of the PROMINENT study with pemafibrate. Adapted from Pradhan et al. [144] with permission. BID, twice daily; HDL, high-density lipoprotein cholesterol; LDL-C, low-density lipoprotein cholesterol; PAD, peripheral artery disease; TG, triglycerides

The PROMINENT study (Pemafibrate to Reduce cardiovascular OutcoMes by reducing triglycerides IN diabetic patiENTs) addresses these critical questions. PROMINENT aims to recruit 10,000 T2DM patients with atherogenic dyslipidemia (TG ≥ 2.3 mmol/L and < 5.6 mmol/L, and low HDL-C) despite statin therapy, with or without established ASCVD (Fig. 7) [144]. Thus, unlike the previous fibrate trials, PROMINENT has specifically targeted the hypertriglyceridemic patient population. The primary endpoint is a four-point MACE of nonfatal MI, nonfatal ischemic stroke, cardiovascular death, or unstable angina requiring unplanned revascularization [144]. The trial is event-driven, requiring 1092 events (at least 200 events in female patients), and is powered to detect an 18% relative risk reduction. Allowing for a placebo event rate of 3.7 per 100 person-years, the trial should take 4–5 years. Within PROMINENT, a prospective nested substudy will investigate whether this SPPARMα agonist slows the progression of diabetic retinopathy in patients with non-proliferative diabetic retinopathy at study enrolment [145]. This substudy follows on evidence of fenofibrate limiting progression of diabetic retinopathy in the FIELD (Fenofibrate Intervention and Event Lowering in Diabetes) and ACCORD studies [146, 147].

Beyond reduction in residual cardiovascular risk, other effects may differentiate this SPPARMα agonist from current fibrates. Notably, pemafibrate can benefit experimental NASH [129], which suggests that this SPPARMα agent can impact progression of complications such as fibrosis, cirrhosis, hepatocellular carcinoma and liver failure [148]. These pathophysiological consequences also extend beyond the liver, contributing to ASCVD burden [149]. Ongoing studies are investigating the effects of this SPPARMα agonist in the setting of NAFLD [150]. Furthermore, combination with a sodium-glucose cotransporter-2 inhibitor may merit exploration, with evidence of favourable effects on weight gain, TG, and glucose levels, and pathogenesis in animals that develop NASH and have heightened risk of hepatocellular carcinoma [151].

## Conclusion

The pandemic of visceral obesity poses enormous socioeconomic challenges in managing the associated cardiometabolic comorbidities of T2DM, NAFLD, and ASCVD. Atherogenic dyslipidemia, chiefly elevated TG-rich lipoproteins and remnant cholesterol (often accompanied by low HDL-C), likely drive this association. There is an unmet clinical need for treatments that effectively reduce residual cardiovascular risk associated with atherogenic dyslipidemia. Realization of the SPPARMα concept and translation to the clinic offers a precision medicine approach to this challenge. On the basis of evidence from preclinical and clinical studies, this Joint Consensus Panel concludes that this SPPARMα represents a new therapeutic class, differentiated from fibrates by its profile of activity, especially improved renal and hepatic safety, as well as lipid-independent anti-inflammatory effects. Consistent with this, the Japanese Atherosclerosis Society has recently ratified SPPARMα as a new therapeutic class, on the basis of these criteria. PROMINENT is testing whether these SPPARMα characteristics translate to reduction in cardiovascular events in T2DM patients with atherogenic dyslipidemia. This study aims to validate SPPARMα as a novel therapeutic class for managing residual vascular risk driven by visceral obesity and T2DM.

Clinical perspective Management of residual cardiovascular risk is evolving to address individual risk characteristics. Global changes in the landscape of cardiovascular risk drivers, specifically increases in visceral obesity and type 2 diabetes mellitus, present an urgent unmet clinical need to manage atherogenic dyslipidemia. Elevated triglycerides, a biomarker of triglyceride-rich lipoproteins and their remnants, characterize this dyslipidemia. Therapeutic approaches have focused on the use of omega-3 fatty acids and fibrates (peroxisome proliferator-activated receptor alpha [PPARα] agonists); however, the latter group have not shown efficacy in improving cardiovascular outcomes in statin-treated individuals, and entail drug interaction and side effect issues, including elevation in liver enzymes, and fenofibrate increases serum creatinine, albeit reversibly. High-dose omega-3 fatty acid did, however, significantly reduce cardiovascular events in REDUCE-IT, justifying the premise of targeting elevated triglycerides.

The development of a selective PPARα modulator (SPPARMα) agonist offers a novel therapeutic approach. Preclinical and clinical studies differentiate the first SPPARMα agonist (K-877, pemafibrate) from current fibrates on the basis of its profile of activity, robust reduction in triglycerides (substantially greater than achieved with omega-3 fatty acid), as well as a favourable safety profile, with no evidence of elevation in serum creatinine. In addition, this SPPARMα agonist may exert more potent anti-inflammatory effects than traditional fibrates. The cardiovascular outcomes study PROMINENT will determine whether therapeutic application of the SPPARMα concept translates to reduction in cardiovascular events in high-risk patients with type 2 diabetes mellitus already receiving the best evidence-based treatment.

## Additional files

**Additional file 1.** Interaction with the selective peroxisome proliferator-activated receptor alpha modulator (pemafibrate, K-877).

**Additional file 2.** Interaction with a PPARα agonist (fenofibrate).

## Data Availability

The datasets used and/or analyzed during the current study (as defined in Box 1) are available from the corresponding author on reasonable request.
